# Comparison of Bagging and Boosting Ensemble Machine Learning Methods for Automated EMG Signal Classification

**DOI:** 10.1155/2019/9152506

**Published:** 2019-10-31

**Authors:** Emine Yaman, Abdulhamit Subasi

**Affiliations:** ^1^International University of Sarajevo, Sarajevo, Bosnia and Herzegovina; ^2^Effat University, College of Engineering, Jeddah 21478, Saudi Arabia

## Abstract

The neuromuscular disorders are diagnosed using electromyographic (EMG) signals. Machine learning algorithms are employed as a decision support system to diagnose neuromuscular disorders. This paper compares bagging and boosting ensemble learning methods to classify EMG signals automatically. Even though ensemble classifiers' efficacy in relation to real-life issues has been presented in numerous studies, there are almost no studies which focus on the feasibility of bagging and boosting ensemble classifiers to diagnose the neuromuscular disorders. Therefore, the purpose of this paper is to assess the feasibility of bagging and boosting ensemble classifiers to diagnose neuromuscular disorders through the use of EMG signals. It should be understood that there are three steps to this method, where the step number one is to calculate the wavelet packed coefficients (WPC) for every type of EMG signal. After this, it is necessary to calculate statistical values of WPC so that the distribution of wavelet coefficients could be demonstrated. In the last step, an ensemble classifier used the extracted features as an input of the classifier to diagnose the neuromuscular disorders. Experimental results showed the ensemble classifiers achieved better performance for diagnosis of neuromuscular disorders. Results are promising and showed that the AdaBoost with random forest ensemble method achieved an accuracy of 99.08%, *F*-measure 0.99, AUC 1, and kappa statistic 0.99.

## 1. Introduction

The neuromuscular system consists of nervous and muscular systems, both of which then compose the human skeletal muscular system. Muscles activities are controlled by the electrical impulses produced by the nervous system. These electrical impulses are identified as motor unit action potential (MUAP). Neuromuscular disorders are caused by different nerves or muscle fibers. Thus, in order to be more focused on the treatment, determination of disorder location is crucial. Electromyography (EMG) is utilized for recording and analyzing the skeletal muscle signals. EMG signals are recorded by inserting electrodes at different location of the muscles. EMG signals are generally employed in clinical applications and human computer interfacing. EMG signals are complex and nonstationary and contain several noises. EMG signals deliver information about the functioning and status of the muscles that can be utilized for the diagnosis of neuromuscular disorders such as myopathy and neuropathy. Neuropathy is a quickly progressive and fatal neuromuscular disorder [1]. It strictly disturbs the functioning of motor neurons. In this disorder, muscles may become smaller and weaker, ultimately resulting in body paralysis [2]. Another muscular disease that involves muscular cramp, stiffness, spasm, and dysfunction is myopathy, which affects skeletal muscles' fiber. Myopathy generally stops muscles to work properly but does not lead to the death of muscles [1, 3]. Diagnosis of neuropathy and myopathy at early phases is difficult since indications of these disorders mimic with that of other disorders. Early detection of these disorders may diminish the suffering of patient and medical costs. It motivates to develop new signal processing-based methods for early detection of these diseases. Quantitative EMG (QEMG) technique which utilizes the quantitative features of EMG signals is an effective method to identify several neuromuscular disorders [1, 4–6]. Conventionally, neuroscientists evaluate neuromuscular disorders based on the MUAP properties [7] and its audio characteristics [8]. Manual evaluation of the anomalies employing the characteristics of MUAPs necessitates skilled and qualified neuroscientists. But the detection of these anomalies by neuroscientists might not be adequate for precise detection of the small variations, and the diverse patterns of the MUAPs cannot be detected easily with manual assessment. Hence, it is essential to carry out analysis of MUAPs, quantitatively, to detect these variabilities in the abnormal patterns. The wavelet transform mainly employed for the analysis of the time series shows nonstationary characteristics [9–11]. The appropriate feature extraction technique is needed for achieving a better classification performance. Neuromuscular disorder detection based on the EMG signal characteristics can be realized either directly [1, 4, 12] or by using MUAP-based methods [2]. In direct method, the EMG signal classification is carried out by dividing the signal into nonoverlapping frames. Then, every frame is employed to extract the features, and eventually, the extracted features are utilized for the classification [3].

Discrimination of EMG signals is crucial to diagnose the neuromuscular disorders. Numerous attributes, such as the quality of the signals, the efficiency of the feature extraction methods and classifiers, and the training and testing datasets, may influence the accuracy of EMG signal classification. Hence, eliminating those factors can enhance the capability of an EMG signal classification system's capability. Recently, several techniques were utilized to obtain efficient features to depict EMG signals for classification [4, 5, 9–11, 13–16]. However, the design of robust and practical computer-aided decision support system is complicated. The complication is to create an accurate and effective decision support system which keeps crucial discriminatory information to achieve better classification accuracy. In this respect, it is necessary to conduct a systematic analysis of EMG signals in order to obtain an efficient classification of EMG. Therefore, some EMG signal analysis algorithms, which are computer-aided, have been developed [4–6, 11, 17–21].

Feature extraction is a technique to extract valuable information that exists in the signals. To classify EMG signals, discrete wavelet transform (DWT) [21] and wavelet packet transform (WPT) [22] based feature extraction methods have been utilized for extraction of features from the EMG signals. In this study, wavelet packet decomposition (WPD), which covers the whole time-frequency plane to provide analysis for low- and high-frequency bands, is used to extract features of EMG signals. After WPD coefficients are extracted, statistical values are calculated for every subbands of WPD. The statistical values of WPD coefficients are utilized as inputs to the classifier. Furthermore, most of the single classifiers are not able to deal with high-dimensional data. Some of the single classifiers can classify high-dimensional data but not working efficiently if the data have a large number of irrelevant variables. In order to eliminate these types of problems, there is a need for EMG signal classification to introduce more efficient machine learning techniques; particularly those can achieve excellent performance. In this paper, to eliminate the restrictions mentioned above, we employed an ensemble classifier framework to classify the EMG signals for the diagnosis of neuromuscular disorders. Furthermore, the performances of ensemble learners have not been compared yet with single classifier algorithms for the EMG signal classification. Hence, this paper analyzes the effectiveness of single classifiers with bagging and boosting ensemble learning algorithms for the EMG signal classification. The main contribution of this paper is to improve general (testing) classification performance by employing bagging and boosting of ensemble classifiers. In order to accomplish better performance, ensemble machine learning methods combine multiple learners' opinions. Thus, better performance could be achieved even by simple learners. In theory, the performance of ensemble classifiers is better than the performance of single classifiers [23–25]. The majority of learners create various feature subsets which is sampled from the original feature data in a random way and utilize voting for ensemble classifiers. Through ensemble learning method, it is possible to use robust classifiers which are used for diagnosis of various disorders. In addition to this, it is possible to conduct various experiments more rigorously by estimating different ensemble classifiers through computer-aided diagnosis (CAD) application. Recently, ensemble classifiers have increasingly gained more attention in CAD applications. Besides, ensemble classifiers have been used for high-dimensional problems which contain extremely different features for instances [26], e.g., microarray data analysis [27, 28] and diagnosis of valvular heart disease [29] for fMRI image data [30]. Furthermore, ensemble classifiers are inherently parallel, so they can be more effective at training and test phases if they can approach multiple processors [31].

The remainder of the paper is organized as follows. In the next section, the literature is reviewed. In Section 3, subjects, feature extraction, dimension reduction techniques, and description of machine learning tools applied for the EMG signal classification are presented.  Section 4 presents a complete experimental work of the proposed EMG signal classification system, in which the effect of feature data and algorithmic matters are studied concerning classification performance. Besides, classification performances of bagging, AdaBoost, and MultiBoosting ensemble classifiers are compared with each other as well as single classification techniques. Finally, Section 5 concludes our work.

## 2. Literature Review

EMG presents comprehensive information to describe the neuromuscular activity and muscular morphology. The EMG signals must be decomposed, classified, and analyzed in order to describe a muscle using quantitative EMG (QEMG) data. In order to diagnose neuromuscular disorders, EMG signals must be classified for the detection of abnormalities [32]. Subasi et al. [33] compared wavelet neural networks (WNNs) with feedforward error backpropagation artificial neural networks (FEBANNs) according to their accuracy for EMG signal classification. Autoregressive (AR) model of EMG signals is utilized as an input to the classifiers. A dataset composed of normal, myopathic, and neurogenic disorder was evaluated. The accuracy of the WNN was 90.7% and that of FEBANN method was 88%. Katsis et al. [34] utilized fuzzy *k*-means for MUAP clustering and then classified the template MUAPs into normal, neuropathic, and myopathic by employing a support vector machine (SVM) classifier. The correct classification success rate is about 86.14%. Kaur et al. [35] showed that SVM classifier achieved 95.90% accuracy for normal, myopathic, and neuropathic EMG signals by identifying the peaks of MUAPs. Rasheed et al. [36] developed a model to distinguish individual MUP waveforms from a raw EMG signal for feature extraction. The adaptive fuzzy *k*-NN classifier achieved 93.5% accuracy with time domain features and 92.6% accuracy with wavelet domain features.

Subasi [4] employed neurofuzzy computing techniques with AR, DWT, and WPE feature extraction methods. The ANFIS classification method yields a classification accuracy of 95% with AR + DWT features. Another study [11] suggested the fuzzy support vector machine classifier united with discrete wavelet transform (DWT) to achieve better performance (97.67% accuracy). Another study [5] proposed a PSO-SVM for the classification of EMG signals in which a set of statistical features were obtained from discrete wavelet transform (DWT) subbands to show the dispersion of wavelet coefficients. Noteworthy improvements regarding classification accuracy were achieved (97.41%). Furthermore, Subasi [10] used an evolutionary SVM classifier for EMG signals classification using normal, myopathic, and neurogenic dataset. In the designed framework, a set of statistical features were obtained from the DWT subbands, and evolutionary SVM achieved an accuracy of 97% using 10-fold cross-validation.

Gokgoz and Subasi [37] studied the effect of multiscale principal component analysis (MSPCA) denoising method in EMG signal classification. Multiple single classification (MUSIC) feature extraction method was implemented on EMG signals to classify into normal, myopathic, and ALS. After denoising with MSPCA, accuracy is 92.55% for SVM, 90.02% for ANN, and 82.11% for *k*-NN. The same researchers [9] presented a framework for EMG signal classification by utilizing MSPCA for denoising, discrete wavelet transforms (DWT) for feature extraction, and decision tree algorithms for classification. The suitable combination of DWT and random forest achieved the best classification accuracy of 96.67% utilizing *k*-fold cross-validation.

Bozkurt et al. [13] employed several parametric methods and subspace-based methods for EMG recordings composed of normal, neurogenic, and myopathic subjects. A combined neural network (CNN) and FEBANN were employed for classification, and the highest performance was achieved with the eigenvector method. The total classification accuracy was 94% for CNN and 93.3% for FEBANN. Khan et al. [38] proposed a framework that utilizes both time domain and time-frequency domain features of the EMG signals. *K*-nearest neighbor (*k*-NN) and support vector machine (SVM) are employed to predict class label (Normal, Neuropathy, or Myopathy) for a given MUAP. DWT-based feature extraction scheme with multiclassifier model achieved 97% accuracy.

Sengur et al. [39] proposed a deep learning-based method for efficient classification of normal and ALS subjects. They used different time-frequency methods combined with the convolutional neural network for EMG signal classification. They employed ALS and normal EMG signals and achieved 96.80% accuracy with CWT and CNN. Hazarika et al. [40] presented a real-time feature extraction and fusion model for automated classification of electromyographic signals with normal, myopathic, and amyotrophic lateral sclerosis using DWT and canonical correlation analysis. The extracted discriminant features are fed to the *k*-NN classifier, and 98.80% accuracy is achieved with two-fold cross-validation. Mishra et al. [41] employed improved empirical mode decomposition (IEMD) in combination with the least-squares support vector machine (LS-SVM) classifier is utilized for the analysis of amyotrophic lateral sclerosis (ALS) and normal EMG signals. The proposed technique is achieved with 96.33% accuracy.

## 3. Materials and Methods

### 3.1. Subjects and Data Acquisition

All measurements of patients and control group were performed by Gaziantep University in Neurology Department. Based on clinical findings, the diagnostic criteria of the selected subjects were also performed by muscle biopsy if necessary. Normal, neurogenic, and myopathic people were evaluated by specialist physicians. The impedance of a concentric needle electrode (0.45 mm diameter with a recording surface area of 0.07 mm^2^; impedance at 20 Hz below 200 kHz) was used to collect EMG signals from the biceps brachii muscle. All signals were collected at 20 kHz for 5 seconds at 12-bit resolution and band-pass-filtered at 5 Hz to 10 kHz. 20 different MUPs were acquired from all muscles in the form of five to seven muscle insertions. Needles between the regions were pulled leastways 5 mm. The acoustic and visual control of the EMG signal was directed close to the active muscle fibers. EMG data were obtained from seven control subjects (three males and four females) with ages in the range from 10 to 43 years (mean age ± standard deviation: 30.2 ± 10.8 years), seven myopathic subjects (four males and three females) with ages in the range from 7 to 46 years (mean age ± standard deviation: 21.5 ± 13.3 years), and thirteen neuropathic subjects (eight males and five females) with ages in the range from 7 to 55 years (mean age ± standard deviation (S.D.): 25.1 ± 17.2 years) as in [4, 5, 11, 15].

### 3.2. Feature Extraction and Dimension Reduction

One of the imperative applications is the capacity to handle data-reduced parameters that are generally named features. Hence, the EMG signals, which consist of numerous data points, can be reduced into a smaller number of features by using different feature extraction and dimension reduction methods. These parameters describe the characteristics of the EMG signals. These methods which use a reduced number of parameters to characterize the EMG signal are crucial to diagnose the neuromuscular disorders. The dimension reduction and feature extraction process composed of two stages:

Stage 1: decomposition of the EMG signals by using the wavelet packet transform

Stage 2: calculation of the statistical values of WPD coefficients

#### 3.2.1. Wavelet Packet Decomposition (WPD)

Wavelets are a group of basic functions of a signal, which is transformed by the wavelet transform. Wavelet achieves a better time and frequency resolution by decomposing a given signal. On the other hand, the wavelets were obtained by dilations and translations from a single function *ψ* [42]. Classes of prime function set between time and frequency are called wavelets. Their symbolization is as follows:

(1) $$\begin{array}{c} \Psi \left(t\right)=\frac{\psi }{\sqrt{S}}*\;\left(\frac{t-u}{s}\right), \end{array}$$

where dilation is symbolized with “*s*” and interpretation parameter is symbolized with “*u*”. During this application, there may be an expansion in the midpoints and the interpretation parameters can be accessed. Signal *x*(*t*) takes correlation operations at discrete frequencies as shown in the following formula:

(2) $$\begin{array}{c} W_{x}\left(u,s\right)=\frac{1}{\sqrt{S}}\int_{-\infty }^{+\infty }x\left(t\right)\psi \left(\frac{t-u}{s}\right)\text{d}t. \end{array}$$

There are many applications that can be seen in literature studies. It is possible to isolate dilation parameters and interpretation parameters of wavelet in a dyadic way. In the following formula, the family/group of wavelets can be seen:

(3) $$\begin{array}{c} \psi_{mk}\left(t\right)=2^{-m/2}*\psi \left(2^{-m}t-k\right). \end{array}$$

The wavelet family is represented as follows. The “*ψ*(*t*)” symbol is the main wavelet. “*m*” represents the dilation parameter, while “*k*” represents translation parameters. The dilation parameter has some responsibilities, which include specifying the wavelet position in the frequency domain, as well as the scale and extent of the time-frequency limitation. The regulation of the wavelets in the third equation can show that each wavelet is orthonormal to another one [43].

Moreover, the WPD uses both the low-frequency components (approximations) and the high-frequency components (details) for the signal decomposition [44–46]. The WPD separates both approximations and details into sublevels in order to realize a better frequency resolution for the decomposed signal. It can be regarded that WPD is a continuous time wavelet decomposition which is sampled at different frequencies at each level or scale. Its advantage is to combine various levels of decomposition for the construction of the original signal [47]. EMG signals are decomposed up to level four in this paper.

#### 3.2.2. Dimension Reduction

When talking about the statistical values, which consist of standard deviation, skewness, and kurtosis, it can be estimated through the use of WPD in order to decrease the signal's dimension. For the signal processing, the first- and second-order statistics are of utmost importance. However, for many signals, especially the signals like EMG, which are nonlinear, statistics of the second order are insufficient. Therefore, for better characterization of signals, statistics of higher order need to be employed as well. Namely, while mean and variance are characterized by the first- and second-order statistics, higher-order moments are characterized by higher-order statistics [47, 48].

Thus, if *X*(*n*) is a random process, one can define the moments of *X*(*n*) as the coefficients in Taylor series expansion of the moment-producing function [47]:

(4) $$\begin{array}{c} \varphi_{x}=\left(w\right)=E\left[\exp\left(jwx\right)\right]. \end{array}$$

If the discrete time signal has zero mean, then the moments are defined as

(5) $$\begin{array}{c} m_{2\;}\left(i\right)=E\left[X\left(n\right),\;X\left(n+i\right)\right],\\ m_{3}\left(i,j\right)=E\left[X\left(n\right),\;X\left(n+i\right)*X\left(n+j\right)\right],\\ m_{4}\left(i,j,k\right)=E\left[X\left(n\right),X\left(n+i\right)*X\left(n+j\right)*X\left(n+k\right)\right], \end{array}$$

where *E*(·) is the expected value of the random process *X*(·) [47].

Taking into account that tools for extraction feature which is based on wavelets feature create the feature vector, the size of which is too huge to be used as an input for the classifier, it is possible to use these dimension reduction techniques to derive a smaller number of features out of the wavelet coefficients. Using the cumulants of first, second, third, and fourth order from each level of subbands, it is possible to calculate the new, reduced, feature sets using the wavelet dissolution subbands. The possibility of translating the set of coefficients into a reduced feature set is one of the most important steps in any classification task. Therefore, this reduced feature set is capable of even better characterizing the behavior of the EMG signal. Consequently, six statistical features are implemented for the classification of EMG signal, and they are as follows [49]:

1. Coefficients' mean absolute values in every subband:

(6) $$\begin{array}{c} \mu =\frac{1}{M}\overset{M}{\underset{j=1}{\sum }}\left|y_{j}\right|. \end{array}$$

(2) Average power of the coefficients in each subband:

(7) $$\begin{array}{c} \lambda =\sqrt{\frac{1}{M}}\overset{M}{\underset{j=1}{\sum }}y_{j}^{2}. \end{array}$$

(3) Standard deviation of the coefficients in each subband:

(8) $$\begin{array}{c} \sigma =\sqrt{\frac{1}{M}}\overset{M}{\underset{j=1}{\sum }}{\left(y_{j}-µ\right)}^{2}. \end{array}$$

(4) Ratio of the absolute mean values of coefficients of adjacent subbands:

(9) $$\begin{array}{c} \chi =\frac{\sum_{j=1}^{M}\left|y_{j}\right|}{\sum_{j=1}^{M}\left|z_{j}\right|}\;. \end{array}$$

(5) Skewness of the coefficients in each subband:

(10) $$\begin{array}{c} \varphi =\sqrt{\frac{1}{M}}\overset{M}{\underset{j=1}{\sum }}\frac{{\left(y_{j}-µ\;\right)}^{3}}{\sigma^{3}}. \end{array}$$

(6) Kurtosis of the coefficients in each subband:

(11) $$\begin{array}{c} \varphi =\sqrt{\frac{1}{M}}\overset{M}{\underset{j=1}{\sum }}\frac{{\left(y_{j}-µ\;\right)}^{4}}{\sigma^{4}}. \end{array}$$

### 3.3. Classification Methods

#### 3.3.1. Artificial Neural Networks (ANNs)

A group of input and output components which are connected creates artificial neural networks. The network acquires the information by adjusting the weights until it can have the ability to anticipate the correct class label of the input tuples. Neurons are connected by weighted links to form a network. Although there are plenty of feasible network structures, the most common one is the multilayer feedforward network. They are interconnected as layers, and it does not exist in either layer connections or cross-layer connections. Each model has an input layer that receives input feature vectors where each neuron generally corresponds to an element of the feature vector. Usually *f*(*x*)=*x* is built for the activation function of the input neurons. The layer which outputs labels is called as output layer, where every neuron corresponds to a label, or a label vector's element. One or more layers can be between input and output layers. These layers are called as hidden layers. Sigmoid function can be used as activation function for functional units like hidden neurons and output neurons [61].

One of the advantages of neural networks is that they have a high tolerance for noisy data. The other advantage is that they can categorize untrained patterns. They might be useful if you do not have enough information about the connections between attributes and classes. They are also intrinsically parallel. So, parallelization techniques might be useful to expedite the process of computation [50].

#### 3.3.2. *K*-Nearest Neighbor (*k*-NN)

The *K*-nearest neighbor classifier, which only stores the training set, is a lazy learning approach because there is no clear training process. It learns by analogy which means the comparison of a provided test tuple with training tuples which are similar. These tuples must be the closest ones to the unknown tuple. A distance metric like Euclidean distance describes the “closeness”. In order to classify *k*-nearest neighbor, the tuple that is not known is selected as the most common class among its *k*-nearest neighbors. The rate of *k* can be determined experimentally [50].

#### 3.3.3. Support Vector Machine (SVM)

SVM method classifies both linear and nonlinear data. A nonlinear mapping is utilized by SVM for converting the primary training set into an upper-level size. SVM examines for the linear optimal separating hyperplane in this new size like a decision border by which the tuples of one class from another are being split. The data from two classes can be separated by a hyperplane which uses a proper nonlinear mapping to an upper dimension. This hyperplane is used to form support vectors that are important training vectors and margins. Contrary to the other methods, they are highly robust for overfitting [50].

#### 3.3.4. Naïve Bayes

Naïve Bayes is one of the probabilistic approaches that utilize semantics in order to represent, use, and learn knowledge. The maximum aposterior (MAP) rule is as follows: an approximation for classifying a test sample *X* is to construct a probabilistic model to estimate the posterior probability *P*(*y*|*x*) of the different *y*'s and to estimate the one with the greatest background probability. In the following formula, Bayes theorem is represented:

(12) $$\begin{array}{c} P\left(y\;|\;x\right)=\frac{P\left(x\;|\;y\right)P\left(y\right)}{P\left(x\right)}. \end{array}$$

In the training set, by counting the proportion of class *y*, *P*(*y*) can be estimated, and while we compare different *y*'s on the same *x*, *P*(*x*) can be ignored. Thus, we only need to consider *P*(*x*|*y*). If we can get an accurate estimate of *P*(*x*|*y*), we will get the best classifier in theory from the given training data, that is, the Bayes optimal classifier with the Bayes error rate, the smallest error rate in theory. However, estimating *P*(*x*|*y*) is not straightforward since it involves the estimation of exponential numbers of joint probabilities of the features. To make the estimation tractable, some assumptions are needed [61].

Naïve Bayes can reach success levels, and it can compete with more sophisticated classifiers. Naïve Bayes' power to detect is undermined because of the dependencies between attributes [50].

#### 3.3.5. REPTree

In a REPTree, a decision tree is made with the help of gain/variance reduction, and the built tree is pruned with reduced error pruning. As it is balanced for speed, just the values for numeric attributes are classified for one time. We are able to set the base number of examples per leaf, maximum tree depth (helpful while tree boosting), least extent of preparing set fluctuation for a splitting (numeric classes), and fold numbers for pruning [50].

#### 3.3.6. LADTree

The LADTree is utilized for two types of class issues through the use of boosting as an alternating decision tree. It is possible to adjust the number of boosting iterations in order to comply with the database and desired complexity-accuracy trade-off. After each iteration, three nodes are added to the tree. One of these nodes becomes a split node, and, if nodes are not combined, the other two become prediction nodes. Even though the alternative algorithms are faster, the specified search algorithm is the most common algorithm. In addition to this, it is possible for the LADTree to adjust the number of boosting iterations so that they will conform to the data. Also, LADTree can adjust the size of the produced tree [50].

#### 3.3.7. C4.5 Decision Tree

C4.5 decision tree method tests for which training examples have the same result are eliminated as they are not very important. Therefore, they are not contained in the decision tree if they do not have minimum two outcomes which have a minimum number of instances. Candidate splits are taken into consideration in the case that they cut a specific number of instances. There is an MDL-based adjustment for splits on numeric attributes. Quinlan [51] designed heuristic in order to avoid overfitting. After that subtraction, we might find out that the information gain is negative. If we do not have attributes that have positive information gain, which is a kind of prepruning, the tree will stop growing. This is indicated at this point since it could be unexpected to get a pruned tree although postpruning is not active [52].

C4.5 employs the gain ratio:

(13) $$\begin{array}{c} P\left(D;D_{1},\ldots ,D_{k}\right)=G\left(D;D_{1},\ldots ,D_{k}\right)\cdot {\left(-\overset{k}{\underset{i=1}{\sum }}\frac{\left|D_{k}\right|}{D}\log\frac{\left|D_{k}\right|}{D}\right)}^{-1}, \end{array}$$

which is a variant of the information gain criterion, taking normalization on the number of feature values. In practice, the feature with the highest gain ratio, among features with better than average information gains, is selected as the split [61].

#### 3.3.8. Random Forests (RF)

The random forest is an ensemble decision tree classifier which has different types of trees. An individual decision tree is produced by using an arbitrary array of features at every node for determining the division. Every tree is based on the values of a random vector taken individually. They have the identical distribution allocated for all trees in the forest. We can form an RF by making use of bagging together with random attribute selection. In order to raise the trees, the CART approach is used. The tree size can be increased to a maximum possible size, and they are not pruned. Random forests make use of random linear combinations from the input attributes. It does not randomly choose a subcluster of the features, but it forms novel attributes which are a linear combination of the existing features [53].

#### 3.3.9. Bagging

Decisions taken from different learners can be combined into one prediction only. Simply combining those decisions in the case of classification is voting. This approach is used by both bagging and boosting. However, the individual models are derived by bagging and boosting in different ways. The same weights are taken by the models in bagging while weighting is given to more successful models in boosting as an executive may put alternative results on a variety of experts' advice relying on their previous correct estimations. The experts are individual decision trees which are made united by making them vote on every test. For a case that one gets more votes than other classes, it is considered as correct. When predictions are made by more number of votes, they are more reliable since there are more voters [52]. Bagging algorithm is shown in Algorithm 1.

#### 3.3.10. Boosting

The boosting is used for combining multiple models to make use of this idea by trying to find models that complete each other. It is similar to bagging in that it exploits voting for the purposes to classify or to average the numeric estimation to single individual model's output. Another similarity is that it brings together models that are of the same type, such as decision trees. On the other hand, it is iterative. While bagging makes use of individual models that are made separately, boosting employs new models that are influenced by the performance of models that were made before. Boosting reinforces new models so that they become experts for instances which are controlled in a wrong way by previous ones. Finally, a model's contribution is assessed by boosting through its confidence not by allocating equal weight to all models [52]. Boosting algorithm is shown in Algorithm 2.

#### 3.3.11. AdaBoost

AdaBoost is a learning algorithm which assumes an instance's weight as a positive number. The existence of instance weights depends on how an error of classifier is measured. It is the total of the weights of the wrongly classified instances separated by the total weight of all instances, instead of the fraction of instances which are wrongly classified. When we weight instances, we may push the learning algorithm to focus on a specific group of instances, which have large weight. There is high importance on such instances since it is vital to classify them correctly. All instances in the training data assigned an equal weight by the boosting algorithm. Then, each instance is reweighted in respect to the classifier's output by the learning method to create a classifier. The weight of misclassified instances is increased, and that of the correctly classified instances is reduced. Hence, easy instances have low weight, and hard instances have a high weight. For the reweighted data, a classifier is created in all subsequent iterations, and that helps correctly classify the hard instances. Depending on the output of this new classifier, the weights of the instances are reduced or increased. So, while hard instances may become harder, easy instances may become easier. However, there are some hard ones which may become easier and vice versa. All of these can be observed in one try. The weights show how frequently the instances have been wrongly classified by the classifiers made till now. At each instance, we form a measure of hardness, and this gives us a good way of producing experts which complete each other. Boosting is better than bagging in producing classifiers which perform better on new data. On the other hand, sometimes it fails in practical situations by producing a classifier that can have smaller success percentage than an individual classifier established from the same database. AdaBoost implies that the combined classifiers fully correspond to the data [52]. AdaBoost algorithm is shown in Algorithm 3.

#### 3.3.12. MultiBoosting

Decision committee learning has shown amazing accomplishment in diminishing arrangement error from learned classifiers. These methods build up a classifier as an advisory group of backup classifiers. The committee individuals are connected to a characterization assignment and their individual yields joined to make a solitary order from committee all in all. This blend of yields is regularly performed by dominant part vote. Since learning aims at building a classifier which is having minimum inaccuracy rate, and if there is no existing information about the data, AdaBoost is an excellent option in standard decision tree learners [54].

AdaBoost and bagging are competent in different mechanisms; if we combine them, we can reach even much better results. Making use of different kinds of approaches in producing committee members is supposed to increase variety in the committee members that may also raise disagreement between predictions. In the case that this could be realized by not considerably increase the error in the individual predictions, it might lower the error in the resulting committee's predictions. Since each type's earlier members have the most significant effect, it could be a good idea to give up on the second members of one type of committee for members that have the efficiency of the initial members of the other type. In MultiBoosting, AdaBoost is combined with wagging, a variant of bagging which fits more to the task than bagging directly. Wagging [55] is a type of bagging which needs a base learning algorithm which can make use of training cases with a variety of weights. Wagging allocates weights randomly to the cases that are present in each training set instead of making use of bootstrap samples in order to establish successive training sets. Gaussian noise was used in Bauer and Kohavi's [55] original formulation of wagging to change the instance weights. That might cause some weights to be lowered to zero, efficiently taking them out from the training set. Aside from the bias and variance reduction features which that algorithm can get each of its constituent committee learning algorithm from, MultiBoosting stands in a better position compared to AdaBoost regarding computation, even though this would demand an alteration to the control of early abortion of learning a subcommittee. The AdaBoost is naturally sequential, which prevents the parallel computation, whereas MultiBoosting in which the classifiers are learned with wagging is independent of the others and makes parallel computation possible for MultiBoosting [56].

## 4. Results and Discussion

**Figure 1:**
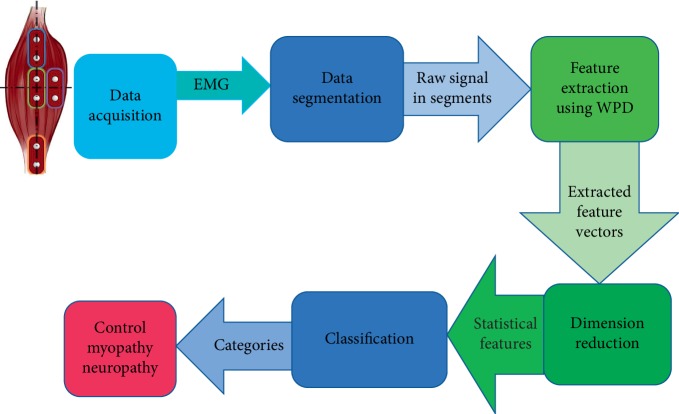
Presentation of the proposed framework.

In this study, for every subband of WPD, the statistical values (first-, second-, third-, and fourth-order moments) were obtained and used for the purpose of classifying EMG signals for diagnosing disorders in the neuromuscular system. Since the number of subjects is limited in this study, the EMG signals are divided into frames with a length of 2048 samples by using rectangular windows. Hence, for each signal class (Normal, Myopathy, and Neuropathy), we have 800 instances, and as a total 2400 instances. The feature of the EMG signal model is described by the derived characteristics from every signal frame. Considering the characteristics of the EMG signal, one can expect the magnitude of a particular feature to vary significantly from person to person. Therefore, it is important to use appropriate classification algorithm which has to tolerate these predicted variations. For this reason, the first step is to acquire a group of features from the EMG signal patterns, and after this, different bagging and boosting ensemble classification algorithms are used for more specified EMG signal recognition as shown in Figure 1.

### 4.1. Performance Evaluation Metrics

Performance on an independent test data does not give a certain scale about the performance of classifiers on the training data. When using a limited number of samples, the classification performance of an algorithm is interesting, and still controversial, one. Hence, in this study, repeated cross-validation which is the option in many applied restricted-data cases is encountered. In cross-validation, a fixed number of folds which are actually partitions of the data are used. Usually, tenfold cross-validation is employed to predict the error rate of a classification method in which a sole, constant example of data is used. Generally, the database is randomly split into ten folds which are called 10-fold cross-validation. Each fold is possessed of almost the same characteristic as the whole database. In the end, a general error prediction is achieved by the average calculation of the ten error predictions [52].

The correct classification results for every class are the true positives (TP) and true negatives (TN), while the false positive (FP) is the result which is incorrectly classified as positive when it is actually negative. Accordingly, the false negative (FN) is the result which is incorrectly classified as negative when, in essence, it is positive [49].

There are parameters are used to calculate the efficiency of the classifier which is recall and precision which can be calculated by using the following formulas:

(14) $$\begin{array}{c} \text{recall}=\frac{\text{TP}}{\text{TP}+\text{FN}},\\ \text{precision}=\frac{\text{TP}}{\text{TP}+\text{FP}}. \end{array}$$

The formula of *F*-measure which is one of the measurement parameters is as follows:

(15) $$\begin{array}{c} F‐\text{measure}=2*\frac{\text{precision}*\text{recall}}{\text{precision}+\text{recall}}=\frac{2*\text{TP}}{2*\text{TP}+\text{FP}+\text{FN}}. \end{array}$$

Finally, of course, the total classification accuracy is as follows:

(16) $$\begin{array}{c} \text{accuracy}=\frac{\text{TP}+\text{TN}}{\text{TP}+\text{FN}+\text{TN}+\text{FP}}*100\%. \end{array}$$

In addition to the above stated, receiver operating characteristic (ROC) curves are a graphical method which is used for the purpose of assessing data mining techniques. This graphical method represents the efficiency of the classifier; however, it does not take into account the class distribution or error costs. [49]. In fact, it is the common way for assessing the area under the ROC curve (AUC) [54–56].

Kappa statistics measure considers this normal figure by deducting it from the indicator's triumphs and communicating the outcome as an extent of the aggregate for an immaculate indicator. The most extreme estimation of Kappa is 100%, and the normal incentive for an irregular indicator with a similar section sum is 0. Kappa measurement is utilized to gauge the understanding amongst anticipated and watched classifications of a dataset while adjusting for an assertion that happens by a shot. In any case, similar to the plain achievement rate, it does not consider the costs [52]. When talking about the statistic which is most often used for evaluation of categorical data in cases where the independent means of assessing the probability of chance agreement between two or more observers are not available, this is the kappa statistic. Namely, a kappa value of 0 represents agreement equivalent to chance, while the kappa value of 1 shows perfect agreement [57, 58]. Cohen [59] defined the kappa statistic as an agreement index and is defined as the following:

(17) $$\begin{array}{c} K=\frac{P_{0}-P_{e}}{1-P_{e}}, \end{array}$$

where *P*_0_ is accepted as an agreement and *P*_*e*_ processes the agreement estimated by chance [60].

### 4.2. Experimental Results

Total classification accuracy, *F*-measure, AUC, and kappa statistics are criteria which are used to assess the accuracy of the single and ensemble learning algorithms. As a result, the outcome of this study has proved that four moments of statistical characteristics obtained for every subband of WPD increase the success rate of classification. The performance of every algorithm was assessed by utilizing the 10-fold cross-validation technique [60]. Classifiers performances for the EMG data are summarized in Tables 1–4. All methods performed reasonably well according to total classification success rate, *F*-measure, AUC, and kappa statistics.  Table 1 shows that LADTree achieved the minimum success rate out of single classifiers' accuracy which is 88.67%. When we checked classification accuracies of ensemble methods, LADTree gave minimum performance with 88.33% (Table 2) in bagging ensemble learning method, and for all of other ensemble learning methods, minimum performance is achieved by NB, 89.54% in AdaBoost method (Table 3) and 88.54% in MultiBoosting method (Table 4). The best performance is achieved by RF with 98.54% classification accuracy from single classifiers. When we used ensemble learning methods, the best performance is achieved by RF with 98.92% in bagging, RF with 99.08% in AdaBoost, and C4.5 with 98.83% in MultiBoosting, respectively.

The *F*-measures of ANN, *k*-NN, SVM, RF, C4.5, Random Tree, REPTree, LADTree, and NB were 0.983, 0.917, 0.978, 0.985, 0.965, 0.951, 0.962, 0.886, and 0.894, respectively, for single classifiers. After using bagging and boosting ensemble learning methods, almost all results are increased until 0.99. The complete performance of these models is demonstrated in Tables 1to 4.

The AUCs of ANN, *k*-NN, SVM, RF, C4.5, Random Tree, REPTree, LADTree, and NB were 0.997, 0.982, 0.986, 0.999, 0.973, 0.963, 0.983, 0.892, and 0.96, respectively. RF achieves the best AUC performance with 1 in bagging and AdaBoost. REPTree achieves the best AUC result with 0.99 in MultiBoosting.

Kappa results for single classifiers ANN, *k*-NN, SVM, RF, C4.5, Random Tree, REPTree, LADTree, and NB were 0.975, 0.875, 0.967, 0.976, 0.947, 0.926, 0.943, 0.83, and 0.843, respectively. After using ensemble learning methods, almost all results are increased until 0.99.

The selection of input variables and classification method choice are the most important considerations for the performance of EMG signal classification. Signal processing method and selection of features are other significant criteria to derive the most valuable parameters from EMG. The best-suited parameters must be used as the inputs of the model for EMG signal classification. For this reason, statistical features extracted for each subband of WPD are chosen and each subband must be relevant to classify the nonlinear dynamics underlying muscle actions and permit foresight into the growth of complication and regularity of the EMG. Diagnosis and treatment of many types of muscle disease are only possible with the exact identification of the EMG signal. Bagging, AdaBoost, and MultiBoosting ensemble learning algorithms were applied to increase the accuracy of single classifiers. As result tables demonstrate, the ensemble classifiers increase the single methods' accuracy in the classification of EMG signals.

### 4.3. Comparison with Previous Studies

Now it is important to compare the efficiency of previous studies with the proposed technique. Accordingly, the accuracy of classification for all studies which were compared is presented in Table 5 which serves to show that the success rate for the technique which we have proposed is higher than the success rate of previous works. It was a challenge to compare the classifiers which were created in this study to those from similar studies, and it was also challenging to compare the diversity of classification methods, MUAP forms which are categorized in systems and the number of MUAP forms classified, and the techniques for EMG signal processing as well as their features. Nonetheless, the results which we have received from this study have a success rate of 99% and are found to perform adequately in comparison with the examples from the literature. Actually, we must compare our results in two ways: one which uses the same dataset and the other which uses different dataset.

A framework formed on the basis of feedforward error backpropagation artificial neural networks (FEBANNs) and wavelet neural networks (WNNs) using the same dataset was suggested by Subasi et al. [15]. For the purposes of EMG signal classification, they also compared the accuracies, and using WNN, they reached the maximum success rate of 90.7%. In [4], 95% of classification accuracy was achieved using the same dataset when ANFIS classifier was utilized with AR and DWT methods for feature retrieval, while in [5], 97% of precision was recorded when DWT feature retrieval and PSO-SVM classifiers were utilized. On the other hand, in [11] DWT was used for the purposes of feature extraction and FSVM for classification, and they reached an accuracy of 97% by using the same dataset. Finally, in [10], the classification accuracy improved to 97.67% due to the use of DWT method for feature extraction and evolutionary SVM for classification. Furthermore, after several years, Bozkurt et al. [13] suggested a model for classifying EMG signal through the use of MUSIC method for feature extraction, combined neural network for classification, and the same dataset. This model was proposed in 2016 and it achieved 94% accuracy. In [21], bagging ensemble with SVM achieved 99% classification accuracy using DWT for feature extraction and the same dataset.

On the other hand, some methods used different datasets as in the study of Katsis et al. [34] who grouped three types of class signal (normal, myopathic, and neuropathic) while utilizing SVM. They achieved 86.14% precision. Additionally, Rasheed et al. [36] introduced an interactive application which serves to implement a classification task during EMG signal decomposition through the use of a fuzzy *k*-NN classifier. Accordingly, they recorded a classification accuracy of 93.5 %. It should also be mentioned that Sengur et al. [39] classified only two class data (normal vs. ALS) through the use of wavelet transform for the purposes of feature extraction and convolutional neural networks (CNNs). The success rate of this study is 96.80%.

When we compare the related studies in the literature with the system we have developed, we see our system performing more successfully than the others. Namely, our proposed technique has the best overall accuracy of 99.08%.

### 4.4. Discussion

In this study, EMG data are applied to design a model to diagnose and cure the neuromuscular diseases. When the dimensions of the EMG signals are very large, it is difficult to work with the data at this size. Therefore, first, the WPD feature extraction technique was applied to extract valuable and informative features. Then, the distribution of wavelet factors is presented by the computing of WPC's statistical numbers. Finally, classifiers utilize the detected feature as entry data. Obtained features were classified with single classifiers firstly. After that, they were classified with bagging, AdaBoost, and MultiBoosting ensemble classifiers, and the results were compared with each other. This study shows that using ensemble learning techniques has improved the success rate significantly. The study of the classification of EMG signals in the literature will reveal that there is almost no study on ensemble techniques in this regard.

In addition to everything above mentioned, it is important to bear in mind that each classification method uses different logic for parameter adjustment. For instance, RF adjusts only one key parameter and that is the number of trees. However, it should be mentioned that the exact meaning of some parameters is still unknown to clinicians. Moreover, since it is difficult to develop a suitable algorithm in clinical practice, scientists have developed numerous machine learning methods. The criteria that are of utmost importance for choosing the suitable algorithm are definitely eased of use, output, and interpretation. Since there is almost no study which employs a method of ensemble learning for establishing a diagnosis of the neuromuscular disorders, this study serves to show the potential that methods of ensemble learning have in establishing a diagnosis of disorders in the neuromuscular system through the use of EMG data.

It was challenging to compare the classifiers which were made in this paper with similar frameworks, diversity of classification methods, the number of MUAP types classified and those classified in the systems, techniques for EMG signal processing, and the characteristics thereof. When compared with examples from the literature, this study has a satisfactory achievement rate of 99.08%. Best result was achieved by the combination of AdaBoost with random forest ensemble method. Random forest is already an ensemble method, the idea is very similar to bagging, it does not use just one algorithm, and it takes decisions from several trees and decides according to mostly voted class. Because of that, it is very strong algorithm. AdaBoost is the most influential boosting algorithm because it minimizes classification error of the combined classifiers. Combination of these two methods gave the best result in our study because of powers of both methods.

When it comes to classifier creation, choice of input variable is crucial. Therefore, as a classifier input, this work utilizes statistic values for each subband of WPC. What enables the smallest subset's usage consistent with the full feature set is the power of statistic features to decrease the number of features in a subcluster. Thus, features linked to signal statistics of diverse frequency bands are the ones which have to be selected for model construction. Consequently, it is of utmost importance that clinicians comprehend the model conditions prior to using it.

## 5. Conclusion

Since the classification of different MUAPs and correct recognition is an important prerequisite for accurate treatment of patients, detection of neuromuscular disorders through the use of electromyography (EMG) recordings has taken a popular and significant place in the area of biomedical research. This study proposes a new framework for characterizing MUAP through the use of statistical values of subband components after wavelet packets (WPD) have been decomposed. In order to remove needless parameters from the main dataset, a simple feature selection method has been suggested. The result of this study serves to show exactly which pathological modifications on EMG signals to the four moments of statistical features are acquired for every subband of WPD shown. It is also important to say that, when compared to similar cases in the area, the suggested method is more exact in distinguishing various MUAP models. Due to the new combination of wavelet packet decomposition with the statistical values, MUAP detection algorithm efficiency indexes are quite satisfactory. Even though there are numerous papers on problems with classification of ensemble learning methods, the number of studies which focus on diagnosing neuromuscular disorders is almost nonexistent. Nevertheless, the results of the study show that the proposed method defeats single classifiers that are most frequently utilized and that the suggested method can be executed in any monitoring application which is computer-based. Best result was achieved by the combination of AdaBoost with random forest ensemble method with 99.08% accuracy. Random forest is already an ensemble method, the idea is very similar to bagging, it does not use just one algorithm, and it takes decisions from several trees and decides according to mostly voted class. Because of that, it is very strong algorithm. AdaBoost is the most influential boosting algorithm because it minimizes classification error of the combined classifiers. Combination of these two methods gave the best result in our study because of powers of both methods. The suggested model uses a small number of parameters representing EMG signals instead of using all EMG records, so using a smaller dataset has given the advantage of performance. Results showed that the AdaBoost with random forest ensemble method achieved an accuracy of 99.08%, *F*-measure 0.99, AUC 1, and kappa statistic 0.99.

## Figures and Tables

**Algorithm 1:**
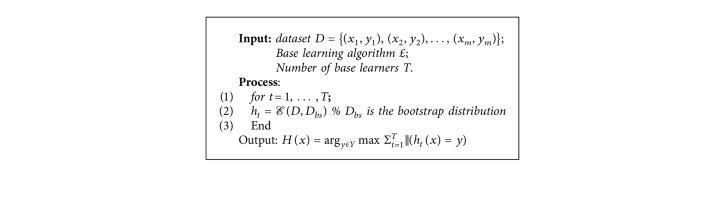
Bagging algorithm.

**Algorithm 2:**
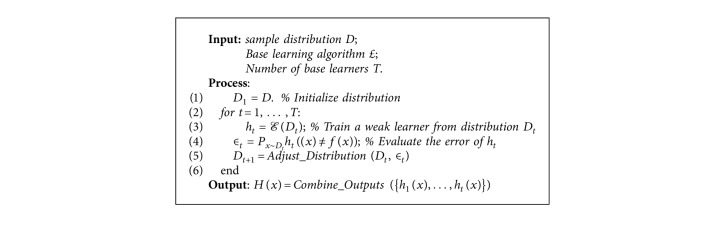
Boosting algorithm.

**Algorithm 3:**
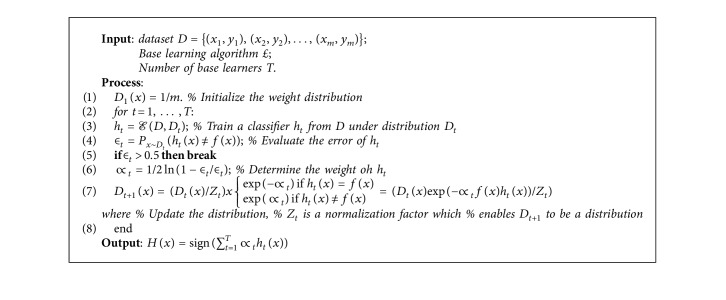
AdaBoost algorithm.

**Table 1:** EMG signal classification results for single classifier.

	Accuracy (%)	*F*-measure	ROC area	Kappa
ANN	98.33	0.983	0.997	0.975
*k*-NN	91.71	0.917	0.982	0.8756
SVM	97.83	0.978	0.986	0.9675
RF	98.54	0.985	0.999	0.9769
C4.5	96.50	96.5	0.973	0.9475
Random Tree	95.13	0.951	0.963	0.9269
REPTree	96.25	0.962	0.983	0.9437
LADTree	88.67	0.886	0.892	0.83
NB	89.54	0.894	0.96	0.8431

**Table 2:** EMG signal classification results for bagging.

	Accuracy (%)	*F*-measure	ROC area	Kappa
ANN	83.33	0.83	0.89	0.81
*k*-NN	91.42	0.914	0.986	0.8712
SVM	98.00	0.980	0.994	0.97
RF	98.92	0.989	1	0.9837
C4.5	98.08	0.981	0.998	0.9712
Random Tree	97.54	0.975	0.997	0.9631
REPTree	97.54	0.975	0.997	0.9631
LADTree	88.33	0.883	0.912	0.825
NB	89.71	0.895	0.968	0.8456

**Table 3:** EMG signal classification results for AdaBoost.

	Accuracy (%)	*F*-measure	ROC area	Kappa
ANN	98.33	0.98	0.99	0.98
*k*-NN	90.50	0.91	0.97	0.86
SVM	97.83	0.98	1.00	0.97
RF	99.08	0.99	1.00	0.99
C4.5	98.88	0.99	1.00	0.98
Random Tree	0.93	0.96	0.95	95.13
REPTree	96.25	0.96	0.98	0.94
LADTree	96.00	0.96	1.00	0.94
NB	89.54	0.89	0.93	0.84

**Table 4:** EMG signal classification results for MultiBoosting.

	Accuracy (%)	*F*-measure	ROC area	Kappa
ANN	98.33	0.983	0.988	0.975
*k*-NN	91.33	0.913	0.976	0.87
SVM	95.88	0.959	0.979	0.9381
RF	98.79	0.988	0.998	0.9819
C4.5	98.83	0.980	0.999	0.999
Random Tree	93.75	0.937	0.953	0.9063
REPTree	98.04	0.980	0.999	0.9706
LADTree	93.92	0.939	0.993	0.9088
NB	89.54	0.894	0.932	0.8431

**Table 5:** Comparison of the classification accuracies achieved by different studies using different datasets.

The study reference	Feature extraction method	Classifier	Classification accuracy (%)
[4]	AR + DWT	ANFIS	95
[5]	DWT	PSO-SVM	97.41
[13]	MUSIC	Combined neural network (CNN)	94
[11]	DWT	FSVM	97.67
[10]	DWT	Evolutionary SVM	97
[15]	AR	WNN	90.7
[21]	DWT	Bagging ensemble with SVM	99
[34]	Fuzzy *k*-means	SVM	86.14.
[35]	Peaks of MUAPs.	SVM	95.90
[36]	Time domain features	Adaptive fuzzy *k*-NN	93.5
[39]	CWT	Convolutional neural network	96.80
Proposed method	WPD	AdaBoost with RF	99.08

## Data Availability

The EMG dataset used to support the findings of this study was supplied by Mustafa Yilmaz under license and so cannot be made freely available. Requests for access to these data should be made to Mustafa Yilmaz (mustafa.yilmaz@gantep.edu.tr).
